# Advances in Antioxidant Applications for Combating ^131^I Side Effects in Thyroid Cancer Treatment

**DOI:** 10.3390/toxics11060529

**Published:** 2023-06-13

**Authors:** Li Yang, Jiahui Ma, Pengyu Lei, Jia Yi, Yilei Ma, Zhongke Huang, Tingjue Wang, Haiyan Ping, Danping Ruan, Da Sun, Hongying Pan

**Affiliations:** 1 Sir Run Run Shaw Hospital, Zhejiang University, Hangzhou 310016, China; 2 Institute of Life Sciences & Biomedical Collaborative Innovation Center of Zhejiang Province, Wenzhou University, Wenzhou 325035, China

**Keywords:** thyroid cancer, ^131^I, oxidative stress, antioxidant, DNA damage

## Abstract

Thyroid cancer is the most common endocrine cancer, and its prevalence has been increasing for decades. Approx. 95% of differentiated thyroid carcinomas are treated using ^131^iodine (^131^I), a radionuclide with a half-life of 8 days, to achieve optimal thyroid residual ablation following thyroidectomy. However, while ^131^I is highly enriched in eliminating thyroid tissue, it can also retain and damage other body parts (salivary glands, liver, etc.) without selectivity, and even trigger salivary gland dysfunction, secondary cancer, and other side effects. A significant amount of data suggests that the primary mechanism for these side effects is the excessive production of reactive oxygen species, causing a severe imbalance of oxidant/antioxidant in the cellular components, resulting in secondary DNA damage and abnormal vascular permeability. Antioxidants are substances that are capable of binding free radicals and reducing or preventing the oxidation of the substrate in a significant way. These compounds can help prevent damage caused by free radicals, which can attack lipids, protein amino acids, polyunsaturated fatty acids, and double bonds of DNA bases. Based on this, the rational utilization of the free radical scavenging function of antioxidants to maximize a reduction in ^131^I side effects is a promising medical strategy. This review provides an overview of the side effects of ^131^I, the mechanisms by which ^131^I causes oxidative stress-mediated damage, and the potential of natural and synthetic antioxidants in ameliorating the side effects of ^131^I. Finally, the disadvantages of the clinical application of antioxidants and their improving strategies are prospected. Clinicians and nursing staff can use this information to alleviate ^131^I side effects in the future, both effectively and reasonably.

## 1. Introduction

**Figure 1:**
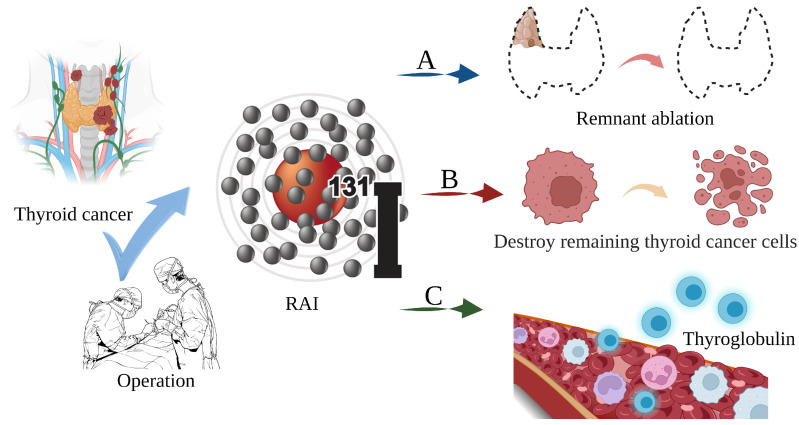
The main role of ^131^I in the treatment of thyroid cancer. (**A**) Thyroid remnant ablation for reducing the likelihood of local recurrence; (**B**) Treating metastatic disease and clearing hidden thyroid cancer cells; (**C**) As a means of addressing persistent disease as reflected by thyroid globulin levels.

Thyroid cancer is a malignant tumor of the endocrine gland that arises from the follicular or parafollicular epithelium of the thyroid gland. As a result of an increased use of diagnostic imaging and surveillance, the incidence of thyroid cancer has been steadily increasing worldwide, with more than 62,000 new cases diagnosed each year [1,2,3]. The most frequent kind of thyroid cancer is differentiated thyroid carcinoma (DTC), which accounts for more than 95% of cases [4,5]. Thyroidectomy, lymph node dissection, and ^131^I therapy are the primary therapeutic options [6]. In clinical practice, ^131^iodine (^131^I), a γ/β radiation radionuclide with a half-life of 8 days, can accumulate in thyroid tissue. As shown in Figure 1, it is commonly used to ablate residual thyroid tissue after surgery (known as thyroid remnant ablation) to reduce the likelihood of local recurrence, treat metastatic disease, and clear hidden thyroid cancer cells [7,8,9]. Iodine-131 is also used as a means of addressing persistent disease as reflected by the thyroid globulin levels [10], with a typical dosage range of 1110 MBq (30 mCi) to 3700 MBq (100 mCi) [11].

However, there is evidence that ^131^I γ/β radiation interferes with the REDOX cell signaling pathways, causing an imbalance between cellular oxidants and antioxidants, resulting in systemic oxidative stress, cell and tissue damage, and an increase in the risk of genetic DNA damage and secondary cancer [12,13,14,15]. Furthermore, it can cause side effects, including salivary gland dysfunction, gastrointestinal reactions, dry eye, pulmonary fibrosis, gonad damage, nasolacrimal duct obstruction, secondary cancer, permanent myelosuppression, and genetic effects [16,17]. To achieve optimal effectiveness and minimize discomfort in thyroid cancer patients, adjuvant medication combinations that reduce the adverse effects of ^131^I are required.

Antioxidants are chemicals that bind free radicals and drastically decrease or prevent substrate oxidation [18,19]. They limit free radical damage by blocking free radicals from damaging lipids, protein amino acids, polyunsaturated fatty acids, and the double bonds of DNA bases [20,21,22]. Notably, substances such as β-carotene and vitamin E have been proven to dramatically minimize the negative effects of ^131^I [23,24]. This review introduces the mechanisms of ^131^I side effects in the treatment of thyroid cancer, focuses on the research progress of antioxidants for reducing the side effects of ^131^I treatment, and proposes the limitations and future trends of antioxidants in the treatment of ^131^I side effects. This information aims to serve as a reference for clinicians, nursing staffs, caregivers, and academies to address the unwanted effects of ^131^I both effectively and reasonably.

## 2. Side Effects of ^131^I

**Figure 2:**
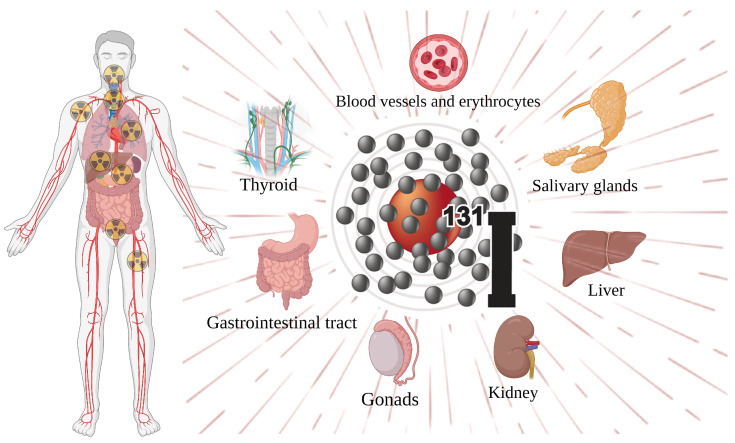
Major body parts affected by the side effects of ^131^I.

Thyroid surgery followed by risk-adapted ^131^I therapy represents the treatment of choice for most DTC patients. In the past, ^131^I therapy was routinely performed to destroy thyroid remnant tissue in low-risk DTC patients with the aim of simplifying the follow-up of such patients by increasing the specificity and accuracy of the basal and/or stimulated Tg measurements. The 2015 American Thyroid Association (ATA) guidelines underscored the role of ^131^I therapy. For low-risk DTC patients, residual ablation is preferred over adjuvant therapy, and a ^131^I dose is recommended at 1110 MBq (30 mCi). However, its use was not indicated or discouraged in low-risk DTC patients (especially those without aggressive features and/or vascular invasion) and in most intermediate-risk cases. Meanwhile, low-risk DTC patients may require adjuvant or even curative ^131^I based on additional risk factors (i.e., patients with additional risk factors or patients requiring maximal treatment) and postoperative assessment (i.e., high postoperative thyroglobulin levels). For intermediate-risk DTC, ^131^I within the range of 1110 MBq to 5550 MBq (30–150 mCi) is usually used for adjuvant treatment. The utility of adjuvant ^131^I treatment in high-risk DTC without identified distant metastasis is noncontroversial due to its high recurrence rate and the improved outcomes with adjuvant treatment [25,26,27]. On the other hand, for the treatment of patients with residual or metastatic DTC, increased amounts of a thyroid-stimulating hormone (TSH) or thyrotropin are required to optimize the selective uptake of radioiodine (RAI) by normal thyroid or cancerous cells. The retention of ^131^I by functioning thyroid tissue is believed to be optimized when serum TSH concentrations are high (30 to 50 μU/mL or more), which can be obtained either by withdrawing levothyroxine (L-T4) or through the administration of a recombinant human thyroid-stimulating hormone (rhTSH) [7,28,29]. Correspondingly, when administered throughout the body, ^131^I remains unavoidably lodged in the bloodstream. The major body parts involved in the systemic side effects are shown in Figure 2 [30,31,32,33,34]. In addition to the most frequent salivary gland diseases, the side effects include genital gland damage, bone marrow suppression, nasal tear tubal obstruction, and dry eye, as well as late sequelae such as persistent osteomyelitis, subsequent malignancy, pulmonary fibrosis, and genetic repercussions.

### 2.1. Salivary Gland Dysfunction

Salivary gland dysfunction is one of the most common complications of RAI treatment, including salivary adenoma, mouth drought, a decrease in or change in taste, and tooth decay symptoms that can appear immediately or months after the treatment of a dose of RAI, and worsen over time [35,36,37]. Salivary glands have an enhanced set of ^131^I through the sodium iodide symporter (NIS) for the physiological iodide intake [38]. The concentration of ^131^I in the salivary gland is approximately 30 to 40 times greater than in the plasma. Acute salivary adenitis is distinguished by saliva gland discomfort and swelling caused by conductor obstruction, mucus retention, and elevated pressure surrounding the conductor [39,40]. Iodine-131 is primarily concentrated in the conductive system, and β radiation can directly damage the salivary gland, causing tubular fragments in the upper cortex of the intralobular ductal epithelium, resulting in conductor blockage, inflammatory reactions in the secretory tissue, and glandular degeneration. In addition, salivary gland stem cells are thought to be mainly present in the excretory ducts. Exposure to β radiation may reduce their regenerative potential and cause damage [41,42]. This damage can lead to endothelial injury and increased vascular permeability, which in turn allows plasma proteins and electrolytes to enter the saliva beyond the usual levels transported by the glandular cells producing sodium and chloride. Consequently, there is an elevation in the sodium and chloride concentrations and a decrease in the phosphate levels in saliva [43]. In addition, many saliva proteins and enzymes have functional and protective effects. Esther N. Klein et al. found a decrease in salivary function 5 months after treatment. A decrease in the saliva flow rate, as well as lower salivary enzymes production, indicates vesicle dysfunction, which can have a long-term cumulative effect on oral health [16,41].

### 2.2. Others

Some organs, such as the breast, digestive tract, and urinary system, concentrate ^131^I whereas others express the NIS, rendering them vulnerable to the impacts of malignant transformation [32]. In addition to early genital gland damage, bone marrow suppression, lacrimal vein blockage, and dry eye disease, the threat of DNA damage to the cells can lead to the accumulation of genetic errors, resulting in genome instability to the extent that it induces late complications that cannot be neglected, including permanent bone marrow inhibition, secondary cancer, pulmonary fibrosis, and genetic effects [22,44,45]. In addition, radiation exposure is a risk factor for the development of secondary malignancies. After ^131^I treatment for thyroid cancer, the incidence of second primary malignancies significantly increases, with the most common being breast and gastrointestinal cancers [32]. According to Fallahi et al., patients receiving a ^131^I activity of more than 37 GBq/1000 mCi have a significantly higher risk of developing second primary malignancies. When the cumulative dose of RAI exceeds 40 GBq (1.08 Ci), the probability of developing second primary malignancies sharply increases [46]. Leukemia incidences significantly increase in patients after RAI therapy and has been found to be more frequent than other cancers [47].

## 3. Oxidative Stress Dominates ^131^I Side Effects

RAI is the standard and effective treatment for DTC. The thyroid gland can accumulate iodine at up to 40 times the concentration of plasma under physiological conditions. This relies on the NIS located in the basolateral membrane of thyrocytes using the electrochemical gradient generated by the Na,K-ATPase as the driving forces that coordinate with the KCNQ1-KCNE2 K^+^ channels located in the basolateral membrane These promote the potassium efflux, thus facilitating iodine transport into the intracellular compartments, and thereby increasing the oxidative stress and cytotoxic efficacy from the radioactivity [39,48,49,50].

**Figure 3:**
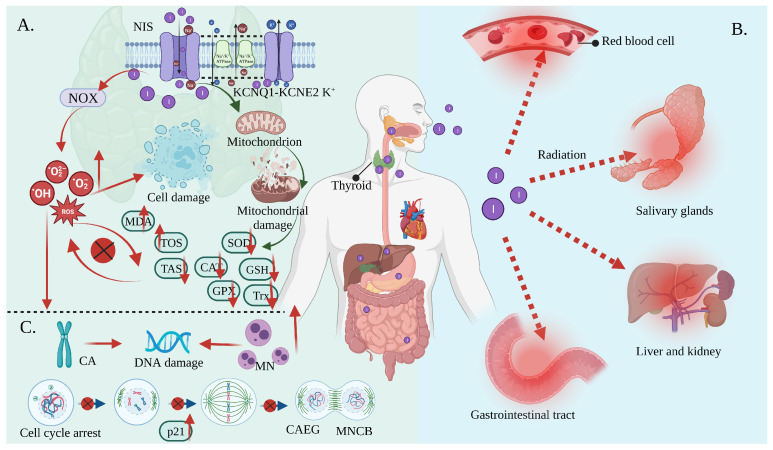
Oxidative stress mediates the side effects of ^131^I. (**A**) Iodine-131 enters the cells through the synergistic transport of the NIS and KCNQ1-KCNE2 K^+^ transporter, and thus increases the expression of NOX1 and changes the ultrastructure of the mitochondria through β/γ radiation, resulting in a reduced antioxidant capacity and the production of numerous ROS. As a result, the activities of CAT and SOD are decreased; the levels of GSH, GPx, Trx, and TAS are decreased; and the levels of MDA and the total oxidative stress (TOS) are increased, leading to systemic oxidative stress. (**B**) Oxidative stress induces erythrocyte membrane damage and vascular permeability changes, salivary gland dysfunction, and gastrointestinal tract and liver and kidney injury. (**C**) Oxidative stress induces a CA and MN increase and mediates a significant increase in the frequency of MNCB, CAEG, and bicentric chromosomes.

Oxidative stress is the result of increased free radical production and/or a decreased antioxidant defense system physiological activity [51,52]. Each cell in a living organism maintains a reductive environment. The reducing environment is maintained by enzymes, which provide constant metabolic energy input to maintain the reducing state [53,54]. This disruption of the normal reduction oxidation (REDOX) state can be mediated by the generation of peroxide-reactive radicals (hydrogen peroxide (H_2_O_2_), superoxide (O_2_^−^), singlet oxygen (1/2O_2_), ROS, and the hydroxyl radical (^∙^OH). The abnormal expression of these substances may result in the destruction of all the components of the cell, resulting in toxic effects [55,56,57]. Severe cases can lead to cell death (Figure 3A). The damage can involve multiple parts throughout the body (Figure 3B).

Iodine-131 can increase the overexpression of NADPH oxidase (NOX)1 in thyroid tissue, resulting in numerous ROS [12]. At the same time, mitochondria are more vulnerable to damage when exposed to iodine radiation. This is due to ultrastructural changes resulting in a decreased antioxidant capacity [58,59]. In other words, the levels of enzymatic antioxidants, including superoxide dismutase (SOD), catalase (CAT), glutathione peroxidase (GPX), and thioredoxin (Trx), as well as non-enzymatic antioxidants such as glutathione (GSH), ascorbic acid, and tocopherol, were reduced in response to ^131^I [60,61]. Herein, ferroptosis described a novel form of regulatory cell death that was induced by fatal lipid peroxidation [62], dependent on iron, which was subsequently induced by an oxidation-damaged phospholipid accumulation and associated with the glutathione-dependent antioxidant defense dysfunction mediated by GPX4 via various pathways. Radiation has been shown to induce ferroptosis [63]. Iodine-131 likely triggered the declines in the metabolism of the lipid peroxides catalyzed by the GPX4 and GSH levels intracellularly and lead to Fe^2+^ oxidizing lipids in a Fenton-like manner, which enhanced ferroptosis and was responsible for thyroid cancer cell death [64]. Comparatively, a GSH deficiency disrupts the REDOX homeostasis, causing ROS accumulation, which eventually results in cell death. The CAT and SOD enzymes play a key role in free radical management, and their reduced activity contributes to an increase in the accumulation of O^2−^ and H_2_O_2_ [65,66,67,68,69,70,71]. Additionally, excessive ROS interact with specific cellular targets to trigger a cascade reaction involving polyunsaturated fatty acid free radicals (lipid peroxidation) on the cell membranes, resulting in an increase in the malondialdehyde (MDA) (marker of lipid peroxidation) levels and a decrease in the CAT, SOD, and GSH activity, resulting in an imbalance between oxidants and antioxidants. The excessive depletion of endogenous antioxidants leads to a decrease in the total antioxidant status (TAS), which ultimately contributes to oxidative stress [70,72,73]. As a result, RAI in the remaining thyroid tissue may result in significant apoptosis and mitotic cell death [74].

In contrast, although most radiation from RAI enters the thyroid gland, a small amount of ^131^I present in the blood and tissues is also capable of causing radiation in other parts of the body [75], such as lipid peroxidation in the kidney, salivary glands, and erythrocytes, resulting in structural and functional damage to the cells [22,75]. Specifically, reductions in salivary TAS, SOD, CAT, and uric acid molecules may have long-term cumulative effects on the oral cavity. A study found that ^131^I treatment decreased SOD activity by 40%. The gastrointestinal tract may be adversely affected as saliva is continuously swallowed after secretion [16]. Other studies have demonstrated that ^131^I ionizing radiation can indirectly promote or induce significant changes in the red blood cell oxidative and antioxidant status. In addition, it can alter the appearance of erythrocytes, as well as their characteristics, such as their lifespan, permeability, and microcirculation [74].

Furthermore, oxidative stress will also involve other aspects, including DNA damage (such as chromosome aberrations (CA) and micronucleus (MN)), changes in the erythrocyte mechanical properties, and changes in vascular permeability. Studies have shown that H_2_O_2_ can induce DNA double-strand breaks and chromosomal rearrangements in thyroid cell lines and primary cultures of human cells (Figure 3C). A significant delay in the repair of γ-radiation-induced DNA damage was observed in human thyroid cells previously exposed to H_2_O_2_ [76]. The studies showing evidence of DNA damage as a consequence of ^131^I treatment are summarized in Table 1. (I) Moreover, ^131^I has been shown to cause transient unstable DNA damage consisting of ROS-induced single-strand breaks and increased chromosomal damage in thyroid cancer patients [13]. (II) The treatment of thyroid cancer using ^131^I (2590 MBq (70 mCi)) caused genetic damage to circulating lymphocytes, with an initial small increase in MDA (1 month to 1.1-fold). The frequency of the binucleated cells that present MN (MNCB) (~1.9 times), aberrant cells excluding gaps (%) (CAEG) (~2.0 times), and double center chromosomes (3.0 times) increased significantly. At 6 months after treatment, there was a further increase in CAEG/dicentric chromosomes but a decrease in MNCB. (III) Ballardin et al. observed a seven-fold increase in the MN frequency after 4 days of RAI treatment (2.96 to 5.50 GBq) in patients, which only reached a baseline after 180 days [77]. (IV) Naoto et al. also reported an increase in MN (3.7 GBq) in patients for a week after treatment [78]. (V) Livingstone et al. observed a six-fold increase in the MN content 11 days after a 9-month continuous treatment (1780 MBq). (VI) Ramabi’rez et al. showed a 2.3-fold increase in MN 1 week after treatment (3700 to 5500 MBq). (VII) Gundy et al. identified an increase in CA in patients treated with ^131^I (1734 to 2600 MBq). (VIII) Baugnet-Mahieu et al. reported a small but significant increase in CA approx. 10 days after treatment (3700 MBq). (IX) M ’Kacher et al. found the presence of persistent biological damage for up to 2 years after treatment exposure using conventional CA assays or chromosome 4 staining [11,75,79,80,81,82,83].

Iodine-131 also altered the transcriptional profiles in another study. Iodine-131 did not induce apoptosis after 24 h, but it increased the p21 levels and prolonged the cell cycle arrest for up to 5 days, indicating that it caused cell senescence. The transcriptome profile of the thyroid cells after ^131^I exposure was similar to that after exposure to H_2_O_2_ and gamma radiation. The thyroid gene expression profiles obtained 4 h after ^131^I exposure revealed a modulation of the *AEN*, *IER5*, *GDF15*, *FAS*, *JUN*, *MDM2*, *CDKN1A*, *BAX*, and *CCL2* expression. These genes have been identified as ionizing radiation response genes in various cell types, including fibroblasts, endothelial cells, and peripheral blood cells, and the thyroid gene expression profiling 24 h after exposure revealed an altered expression of the genes involved primarily in cell division, mitotic/cell cycle regulation, apoptosis, and DNA repair [85].

## 4. Antioxidants Reduce ^131^I Side Effects

**Figure 4:**
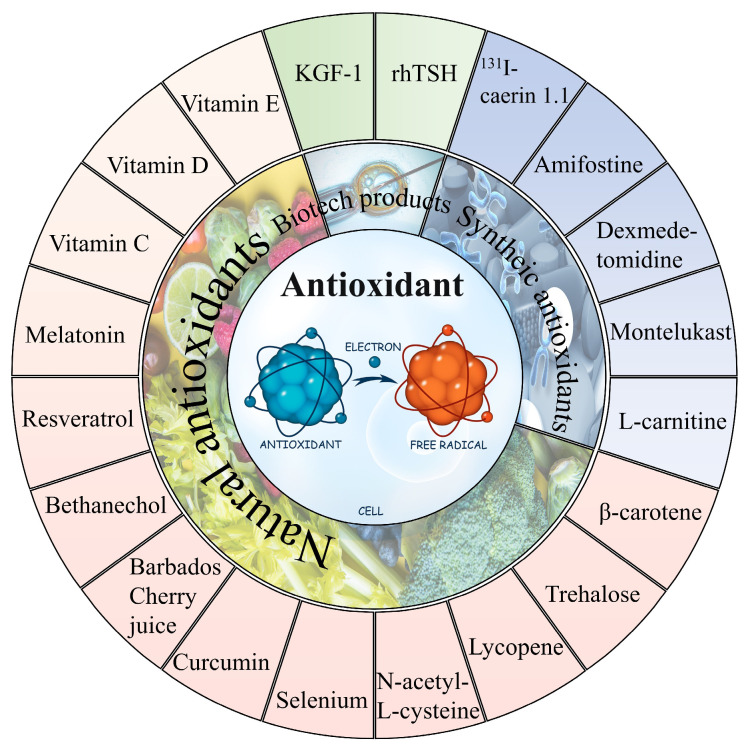
The natural and synthetic antioxidants applied to combat ^131^I side effects.

In general, it can be observed that oxidative stress mediates the pathological process of almost all the ^131^I side effects. Herein, the antioxidants showed a robust effectiveness against their side effects. The antioxidants that have been proven to alleviate the side effects of ^131^I are shown in Figure 4 and the drug type, drug treatment, subject, dose, side effects, and drug efficacy are summarized in Table 2.

### 4.1. Natural Antioxidant

Natural antioxidants, sourced mostly from plants, counteract radiation by neutralizing the free radicals produced in the body when it is exposed to the radiation [98,99]. The mechanism of action generally involves scavenging free radicals and preventing them from damaging cells, tissues, and DNA. As a result, they are capable of shielding the organism cells from damage and aiding in the prevention of cancer and other health problems associated with exposure to radiation [100]. One of the advantages of natural antioxidants is that they are safer than synthetic antioxidants and have been utilized in conventional medicine for centuries. Furthermore, natural antioxidants are metabolized by the body into harmless compounds, most of which are excreted through normal metabolic processes and are more easily tolerated [101,102].

Vitamin C as ascorbic acid regulates the activity of the glutamate receptors, lowering the level of free radicals produced by the glutamate release, and has been proven to reduce the frequency of chromosomal aberrations by approximately 30%, significantly reduces the number of DNA breaks, and has a repairing effect on DNA [103,104].Vitamin C reacts directly with alkoxyl, hydroxyl, and lipid peroxyl radicals or neutralizes them and converts them into water, alcohols, and hydroperoxylated lipids, respectively. Importantly, studies have indicated that vitamin C has a radioprotective effect against oxidative stress, regardless of the timing of administration before and after RAI treatment [43]. Vitamin C in plasma leads to an increased resistance to lipid peroxidation and a decrease in DNA, lipid, and protein oxidation. In addition, vitamin C leads to the neutralization of free radicals of other antioxidants in the form of glutathione and vitamin E, as well as their regeneration. Approx. 2 days after RAI (5550 MBq), the MDA levels and CAT activity declined and the GSH levels decreased, while the daily administration of 1500 mg vitamin C starting two days before significantly reduced the MDA levels and not only prevented the reduction in GSH, but also significantly increased its levels after RAI treatment [22].

Additionally, vitamin E is the collective term for four tocopherols (α-, β-, γ-, and δ-tocopherols) and four tocotrienols (α-, β-, γ-, and δ-tocotrienols) found in food, and is a lipid-soluble antioxidant that protects polyunsaturated fatty acids in the membranes from oxidation, regulates the production of reactive oxygen species and reactive nitrogen species, and modulates the signal transduction [73]. The significant protective effect of vitamin E on the parotid and submandibular glands after ^131^I (23 mCi) treatment with DTC has been published [87,105], which was comparable to the results of Filiz Aydoğan et al. [106]. RAI (111 MBq/kg) resulted in a significant increase in the tissue TOS, TNF-α, IL-6 levels and a significant decrease in the IL-10 and TAS levels, while vitamin D (200 ng/kg/day) dramatically reversed all these parameters [88]. Meanwhile, sialogogues such as lemon candy, vitamin E, lemon juice, and lemon slices as well as parotic gland massages may all minimize injury to the salivary glands [10]. Parotid massages, aromatherapy, vitamin E, selenium, and bethanechol showed a significant reduction in the salivary gland dysfunction induced from the ^131^I treatment (2960–7890 MBq) [43]. Additionally, keratinocyte growth factor-1 (KGF-1) (100 μg/1 mL PBS) restored saliva homeostasis and reduced the ^131^I-induced (0.01 mCi/g) cell apoptosis in the mice [90]. A marker of lipid peroxidation, 8-Epi-PGF_2α,_ is the outcome of free radical-mediated arachidonic acid peroxidation, and the effect of high-activity treatment (2960 or 7400 MBq) is significantly higher and longer in length than that of low-activity treatment (185 or 740 MBq), with a dose-dependent oxidative damage in vivo [107]. In the research of Rosário et al., the 8-epi-PGF_2α_ concentrations were significantly higher in thyroid cancer patients 2 days before and 7 days after the ^131^I injection, and the increase (percentage) was significantly larger (mean 112.3% vs. 56.3% compared to the intervention group). Iodine-131 (3.7 GBq) after 2 days of plasma 8-epi-PGF_2α_ significantly increased, while the daily intake of 2000 mg of vitamin C, 1000 mg of vitamin E, and 400 µg of selenium for 21 days before RAI treatment significantly reduced 8-epi-PGF_2α_ and inhibited oxidative stress [86].

In terms of the protection against DNA damage, the use of curcumin and alginate as antioxidants reduced the number of DSBs caused by ^131^I. At the same time, the radiation protection effect of curcumin exceeded that of trehalose [84]. Melatonin and Se NPs (as radioprotective agents) reduced the ^131^I-induced DSBs levels in peripheral lymphocytes [90]. Vitamins E and C were capable of reducing the DSBs levels by 21.5% and 36.4%, respectively [23]. The positive results of the Barbados cherry fruit radiation protection may be due in part to its rich content of antioxidant compounds, including vitamins A, B1, B2 and C; carotenoids; anthocyanins; phenols; and flavonoids. The ^131^I (25 μCi) treatment of Wistar rats with an increased thyroid function and associated vitamins and sugars from the Barbados cherry fruit stimulated a significant increase in the mitotic index in the normal cells of the rat bone marrow. In particular, the Barbados cherry juice (5 mg) may act as an effective scavenger of the reactive oxygen species in acute radiation protection treatment, protecting the cells by neutralizing free radicals before and during treatment. Meanwhile, it may play a role in the healing process of ionizing radiation-induced damage after treatment. Barbados cherry sub-chronic treatment has higher radioprotective activity in terms of trapping free radicals or preventing their formation [91]. N-acetyl-L-cysteine has also been demonstrated to guard against an increase in ROS and eventual DNA damage in thyroid cells caused by ^131^I in vivo [92]. Before, during, and after ^131^I treatment, β-carotene exerts a significant anti-mutagenic/radioprotective activity, stimulates the DNA repair systems, and minimizes chromosomal aberrations and genetic material damage [12]. Apart from this, resveratrol had anticancer and antioxidant effects, protected the histopathological pattern of the lacrimal gland from damage, reduced inflammation in the histopathological assessment, and decreased the histocytokine levels, apoptosis, and DNA fragmentation on the lacrimal gland after RAI [93]. Iodine-131 caused an edema of the duodenum and ileum lamina propria, duodenal ulceration, gastric mucosal erosion, and gastric and colonic mucosal degeneration in the rats, whereas lycopene resulted in a statistically corresponding reduction in the inflammation present [94].

### 4.2. Synthetic Antioxidants

Synthetic antioxidants have advantages in radiation protection due to their greater potency, consistency, stability, and application flexibility. Despite the fact that natural substances have been used in traditional medicine for centuries, their variability, lack of specificity, and instability require modifications to their properties [108,109]. Accordingly, synthetic substances offer a reliable and effective way to protect against the harmful effects of radiation. Thus, further research and development is required to create more effective radiation protection, safer synthetic substances for human consumption, and to determine the safe limits for their applications [110,111,112]. However, it is important to note that synthetic antioxidants can frequently cause adverse health effects when used in high doses [113].

Iodine-131 (555–660 MBq) treatment with 200 mg/kg L-carnitine or amifostine for 10 days can provide radiation protection and reduce salivary gland injury [34]. Amifostine is an organic thiophosphate, which is dephosphorylated to the active metabolite WR-1065 in normal tissues. Once activated in the cells, WR-1065 acts as a free radical scavenger. Additionally, many studies have reported the radiation-proof effect on ^131^I treatment [35,114].

Iodine-131 causes transient unstable DNA damage composed of reactive oxygen-induced SSBs, and increased chromosome damage in hypothyroidism patients (mutations in enzymes deputed to DNA repair (DNA-1) or in the enzymes involved in the scavenging of free oxygen radicals (DNA-2)). The rhTSH administration reduced radiation exposure by 27% over 120 h and decreased the genomic instability by maintaining hyperthyroidism and normal renal clearance (Epi-GFR and creatinine values). It significantly induced a reduction in the reactive oxygen metabolites-derived compounds. The patients had less radiation-induced chromosome damage, even though several enzyme mutations were present [13].

Lin et al. prepared a drug delivery system with ^131^I-labeled caerin 1.1 peptide (F1) (200 μCi ^131^I and 8 μg caerin 1.1 peptide). The MTT results showed that 5 μg F1 had an inhibitory effect on the CAL-62 cells cultured in vitro. Interestingly, studies identified weight loss over time in the ^131^I treatment group in vivo, but not in the ^131^I-F1 or F1 groups. It is possible that ^131^I-F1 or F1 was confined to the tumor after injection, while ^131^I may have entered the microcirculation through the blood vessels within the tumor and then entered the internal circulation. In view of the fact that radiation entering the human body can cause acute injury, the occurrence of acute radiation sickness or syndrome characterized by weight loss suggests that ^131^I-F1 is safer with fewer side effects [96].

Additionally, synthetic drugs have been studied for the treatment of other side effects. Treatment with dexmedetomidine (3 μg/kg) significantly decreased the levels of MDA, advanced the oxidized protein products induced by RAI (2 MBq), significantly increased the levels of the total sulfur group and CAT, and reduced histopathological abnormalities, which could be applied as a post-^131^I liver protection regimen [97]. In the case of RAI, a high absorbed dose may be produced in the lung parenchyma, thus causing lung damage [115]. Montelukast (10 mg/kg/day) significantly reduced the degree of inflammation and pulmonary fibrosis in the Wistar rats treated with ^131^I (111 MBq/kg). The authors attributed this protective effect in part to the antioxidant effect of montelukast [45].

### 4.3. Antioxidant Deficiency

In summary, the application of the above antioxidants will hopefully play an important role in alleviating the side effects of ^131^I. It is important to highlight that even when the use of antioxidants has been shown to ameliorate the side effects of ^131^I therapy, there are also reports on the drawbacks of using them. Some antioxidants induce oxidative stress at high concentrations (e.g., β-carotene) [24]. Meanwhile, it has been reported that an excessive vitamin E intake can affect the absorption and function of other fat-soluble vitamins [116]. Furthermore, synthetic antioxidants have been reported to cause potential health hazards, including liver damage and cancer [117,118,119]. Therefore, further investigation is needed at a pre-clinical level to standardize the use of antioxidants as adjuvants for ^131^I treatment.

## 5. Challenges and Prospects

**Figure 5:**
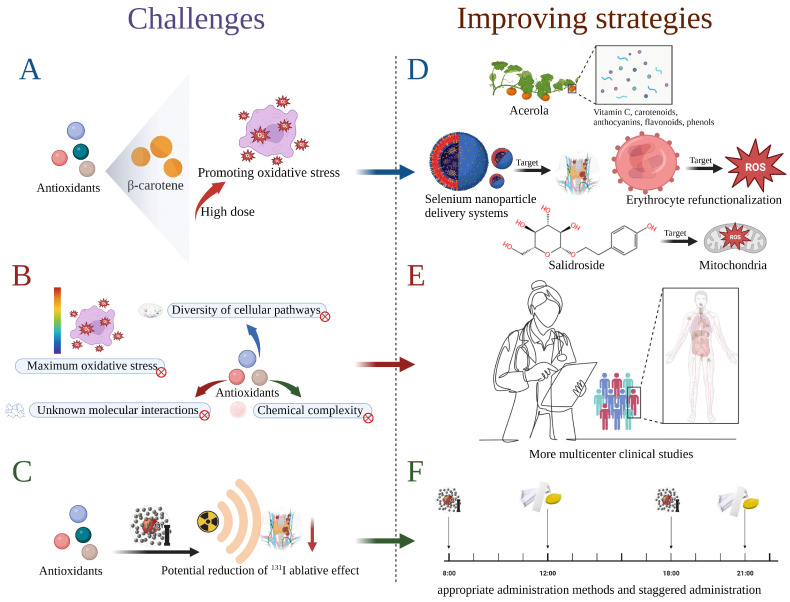
The challenges for the clinical application of antioxidants combating ^131^I side effect: (**A**) High concentrations of antioxidants (such as β-carotene) may promote oxidative stress. (**B**) Research on some antioxidants is incomplete, including diversity of cellular pathways, maximum oxidative stress, unknown molecular interactions and chemical complexity. (**C**) There may be potential risks associated with the improper use of antioxidants. And the corresponding improving strategies are as follow. (**D**) Consume vegetables containing a variety of antioxidants or develope NPs applications. (**E**) Conduct more multicenter clinical studies. (**F**) Seek appropriate administration methods and staggered administration according to the nature of the drug.

Notably, the clinical use of antioxidants presents the following challenges shown in Figure 5A–C. (A) Studies have shown that, at high concentrations, beta-carotene may have agonistic activity (i.e., pro-oxidant) and may induce oxidative stress by increasing free radicals or failing to reduce the mutagenicity of ^131^I ionizing radiation [24]. (B) Some antioxidants have a complex mechanism of action that is not fully understood. For instance, studies have found that vitamin C and pilocarpine do not have a significant protective effect against salivary gland dysfunction [43]. A daily dose of 1500 mg of vitamin C in thyroid cancer patients 2 days after surgery did not significantly alter the GSH levels, and its role as a oxidative stress reliever is questionable [22]. In other words, some of the chemical complexity of antioxidants, the diversity of cellular pathways that may be involved, and their interactions with other molecules in the cell remain to be studied. Bartoc et al. identified that the plasma total antioxidant capacity decreased significantly after ^131^I treatment for 1 week. In this study, the TAS showed no significant difference between 1 month and 6 months after treatment, which may indicate that the maximum period of oxidative stress was missed and the recovery period had already been entered [11]. (C) A potential risk associated with the use of antioxidants is that they may reduce the ablative effect of ^131^I, since its efficacy is dependent upon radioactivity.

The corresponding potential strategies are as follows (Figure 5D–F). (D) Consuming vegetables containing a variety of antioxidants, such as acerola, which contain vitamin C, carotenoids, anthocyanins, flavonoids, and phenols, may be more beneficial than eating individual synthetic carotenoids [24]. Encouragingly, nanoparticles (NP) (mainly mesoporous silica, gold, carbon, or liposomes) have been developed to carry drugs with high payloads, prolong the half-life of drugs, reduced toxicity of the drugs, enhance the solubility of drugs, increased the targeting efficiency, finetune the pharmacological properties, and thereby improve the detection of biomarkers and routine laboratory parameters (e.g., thyroid-stimulating hormone, thyroglobulin, and calcitonin), tumor imaging, and drug delivery in TC [120]. Drug loaded nanocarriers for the treatment of anaplastic thyroid cancer have been developed to address the abnormal expression of the NIS, as current treatment methods are suboptimal [121,122]. Li et al. developed lipid-peptide-mRNA NPs capable of adsorbing an mRNA encoding NIS, which can increase the NIS expression in anaplastic thyroid cancer cells more than 10-fold and result in a higher ^131^I accumulation in the tumor [123]. Further, Zou et al. successfully prepared selenium nanoparticle delivery systems FTY720@T7-SF-Se NPs (silk fibroin (SF), selenium nanoparticles (Se NPs), fingolimod (FTY720), and heptathiepin (T7)), which enhanced the permeability and retention of the tumor sites [124]. Nanospheres can serve as an effective treatment for thyroid cancer and also provide a new idea for how to resolve the negative effects of ^131^I. At the same time, the re-functionalization of red blood cell-based nanomaterials to enhance the targeted drug delivery strategy at the site of oxidative stress injury can also be considered as a key reference [125]. In addition, salidroside has been identified as a mitochondria-targeted antioxidant to prevent salivary gland damage caused by X-ray radiation [126]. The development of drugs that target ^131^I-damaged organs and tissues may provide an alternative solution to the side effects associated with high doses of traditional antioxidants. On the other hand, it is necessary to fully study the temporal and spatial distribution of the oxidative stress state in various parts of the body for thyroid cancer patients after ^131^I treatment, and then develop a personalized combination therapy of antioxidants based on the drug pharmacokinetics, patients’ disease status, and other factors that may affect the duration of the medication. (F) It is important to highlight that more investigation is needed at a pre-clinical level to standardize the use of antioxidants as adjuvants of ^131^I treatment. This requires researchers to conduct further high-quality, multicenter clinical studies that can help standardize treatment protocols and harmonize measurement techniques to ensure research consistency and produce reliable results. (G) It is worth referring to measures similar to lemon candy, sugar-free gum, etc. for the prevention of salivary gland damage (similar targeting) [127]. Using a cross-peak administration approach, antioxidant supplements can be taken a few hours after RAI therapy or during the rest period between treatments.

## 6. Conclusions

Incidences of thyroid cancer, primarily DTC, continue to rise. Iodine-131 plays an excellent role in assisting the ablation of residual cancer cells in vivo after surgery. However, since ^131^I accumulates in normal tissues except the thyroid, radiation damage is brought about to multi-organ tissues as a result of oxidative stress. Both natural substances and synthetic antioxidants can restore cell function by scavenging ROS free radicals, maintaining the oxidant/antioxidant balance in the body, and reducing DNA damage, with positive responses to thyroid damage, salivary gland dysfunction, dry eye, pulmonary fibrosis, gonad damage, nasolacrimal duct obstruction, gastrointestinal reaction, and other side effects. Several challenges, including some antioxidants, probably induce oxidative stress at high concentrations (e.g., β-carotene). The low targeting and unclear mechanisms of antioxidants in practical application can also be addressed through higher-quality multicenter clinical studies, the search for targeted drugs at sites of oxidative stress, or the development of delivery systems based on the re-functionalization of erythrocytes. It is believed that the administration strategy of ^131^I supplemented with antioxidants can provide a reference for clinicians, nursing staff, caregivers, and academics to alleviate the side effects of ^131^I in the future, both effectively and reasonably.

## Figures and Tables

**Table 1:** DNA damage caused by ^131^I. (single-strand breaks (SSBs); double-strand breaks (DSBs); anti-reactive oxygen metabolites (Anti-ROMs); reactive oxygen metabolites-derived compounds (d-ROMs)).

Subject	Dose of ^131^I	Test Site	Side Effects of ^131^I	Ref.
Thirty-one patients in hypothyroidism (HYPO group) and 31 patients in euthyroidism (rhTSH group)	1850 MBq	blood	In the HYPO patients, the radiation exposure rate, chromosome breaks, SSBs, DSBs, total exchanges (DNA-1), transient unstable DNA damage, stable DNA damage, anti-reactive oxygen metabolites (Anti-ROMs), “FAST” antioxidants (Anti-ROMsF), polymorphisms, DNA mutation score↑ and d-ROMs, “SLOW” antioxidants (Anti-ROMsS)↓ at one week. d-ROMs and Anti-ROMsS↑ at 3 months compared to one week.	[13]
Nineteen patients (16 women and three men) suffering from thyroid cancer	2590 MBq	blood	MN and CA↑ and the in-serum uric acid concentration↑ after 1 month. Thiobarbituric acid-reactive products↓ after 6 months.	[11]
Eleven patients already submitted to total thyroidectomy	Between 2.96 and 5.50 GBq	peripheral blood lymphocytes	MN and clastogenic factor↑	[77]
Twenty-two DTC patients	3.7 GBq	Peripheral blood lymphocytes	MN↑	[78]
Ten patients suffering from thyroid cancer	1850 MBq	Circulating blood lymphocytes		[75]
A 34 year old male patient	1780 MBq	Lymphocytes		[80]
Twelve women with papillary or folhcular thyroid cancer	3700–5500 MBq	Blood lymphocytes	Clastogenic effects, X chromosome-independent aneugenic activity↑ at 1 week after treatment.	[81]
Fifty DTC patients	3.7 GBq	Peripheral lymphocytes	CA↑ approx. 10 days after treatment	[82]
Nineteen DTC patients	1734–2600 MBq	Blood lymphocytes	CA↑	[84]

**Table 2:** The applications of various antioxidants to alleviate the side effects of ^131^I. (8-Epi-prostaglandin F2alpha (8-epi-PGF2α); uptake fraction (UF); uptake index (UI); excretion fraction (EF); excretion ratio (ER); first-minute uptake ratio (FUR); maximum uptake ratio (MUR); hypoxia inducible factor-1α (HIF-1α)).

Drug Type	Drug Treatment	Subject	Dose of ^131^I	Side Effects of ^131^I	Drug Efficacy	Ref.
Natural antioxidant	Daily supplementation consisting of 2000 mg vitamin C and 1000 mg vitamin E and 400 µg selenium for 21 days before ^131^I	Forty patients with thyroid cancer submitted for thyroidectomy (n = 20)	3.7 GBq	8-epi-PGF_2α_↑	8-epi-PGF_2α_↓	[86]
	1500 mg vitamin C daily 2 days after (group 2), 2 days before to 2 days after (group 3), and 2 days before RAI (group 4)	Fifty-eight DTC patients ablated with ^131^I	5550 MBq	MDA, CAT↑; GSH↓	MDA↓ (group 2,3,4); GSH↑ (group 3,4); CAT↓ (group 3,4)	[22]
	Groups A, B, and C received vitamin E 100, 200, and 300 mg/day orally, respectively, for a duration of 1 week before to 4 weeks after I therapy	Eighty-two DTC patients with ^131^I	100 mCi	UF, UI, EF, and ER↓	UI, EF, UF, ER↑	[87]
	Vitamin D (200 ng/kg/day)	Wistar albino rats (n = 12)	111 MBq/kg	TOS, TNF-α, IL-6↑; IL-10, TAS↓	TOS, TNF-α, IL-6↓; IL-10, TAS ↑	[88]
	Vitamin E (800 IU/day for one week before and four weeks after RAI therapy)	Thirty-six DTC patients with RAI (n = 18)	3700–5550 MBq	FUR, MUR, MSP, and EF↓	FUR, MUR, MSP, and EF↑	[43]
	Bethanechol (2 mg orally twice a day) for one month after ^131^I	Fifty DTC patients with RAI (n = 25)	97.2 to 213.4 mCi	MUR, MSP, ΔMS, EF↓	Serum amylase↓	
	Selenium 300 mcg orally for ten days (from three days before until six days after RAI therapy)	Sixteen DTC patients with RAI (n = 8)	3.7 GBq	Xerostomia, sialadenitis symptoms↑	Xerostomia, sialadenitis symptoms↓	
	KGF-1 (100 ug/1 mL PBS)	Eighteen C57BL/six mice (n = 6)	0.01 mCi/g	HIF-1α↑; mucin stained acini, amylase↓; periductal fibrosis↑	HIF-1α↓; mucin stained acini, amylase↑; periductal fibrosis↓	[89]
	50 μg curcumin per mL of blood and 5.738 mg trehalose per mL of blood	Blood of five humans	20 μCi	DSB increased to 102.9%	DSBs decreased by 42% (curcumin) and 38% (trehalose)	[84]
	0.0167 mg melatonin per mL of blood and 0.025 mg Se NPs per mL of blood	Blood of five humans	20 μCi	DSB increased to 102.9%	DSBs decreased by 38% (melatonin) and 30% (selenium nanoparticles)	[90]
	0.0666 mg vitamin E per mL of blood and 0.0167 mg vitamin C per mL of blood	Blood of five humans	20 μCi	DSB increased to 102.9%	DSBs decreased by 21.5% (vitamin E) and 36.4% (vitamin C)	[23]
	Barbados Cherry juice (5 mg)/100 g	Wistar rats (n = 6)	25 μCi/100 g	1,1-diphenyl-2-picrylhydrazyl↑; chromosomal and cellular aberrations↑	1,1-diphenyl-2-picrylhydrazyl↑; chromosomal and cellular aberrations↑	[91]
	20 mmol N-acetyl-L-cysteine	Normal differentiated rat thyroid cell line PCCL3	10 μCi/mL	ROS, DBS, MN↑	ROS, DBS, MN↓	[92]
	8 mg β-carotene/mL corn oil (0.2 mL/100 g)	Wistar rats (n = 6)	25 μCi /100 g body weight	CA, MN, water consumption↑	CA, MN, water consumption↓	[12]
	20 mg/kg/day resveratrol	Thirty Wistar albino rats (n = 10)	3 mCi/kg	Caspase-3, TUNEL, TNF-α, IL-6, nuclear factor-kappa-B (NF-кB), TOS↑; IL-10, TAS↓	Caspase-3, TUNEL, TNF-α, IL-6, NF-кB, TOS↑; TAS↓	[93]
	1 mL lycopene (5 mg/kg body weight)	Twenty Wistar albino rats (n = 10)	3 mCi	Duodenal and ileal lamina propria edema, duodenal ulcer, gastric mucosal erosion, and gastric and colon mucosal degeneration↑	Duodenal and ileal lamina propria edema, duodenal ulcer, gastric mucosal erosion, gastric and colon mucosal degeneration↓	[94]
Synthetic antioxidants	200 mg/kg amifostine or L-carnitine	Forty adult guinea pigs	555–660 MBq	Body weight and thyroid hormone↓	Body weight and thyroid hormone↑	[34]
	200 mg/kg amifostine to three rabbits/500 mg/m^2^ amifostine before ^131^I to eight patients	Five rabbits/17 patients	1 GBq to rabbits/6 GBq to patients	Reduced parenchymal function in parotid and submandibular glands; xerostomia; lipomatosis	None of the parenchymal function in parotid and submandibular glands reduce, xerostomia and lipomatosis occurred	[95]
	rhTSH (1 mg/2 d and 1 mg/1 d before ^131^I)	Sixty-two patients prepared with rhTSH or by thyroid hormone withdrawal	1850 MBq	CA, MN, ROS↑	CA, MN, ROS↓	[13]
	8 μg of F1 peptide labeled with 200 μCi ^131^I every 3 days for a total of three times	Nude mice with human anaplastic thyroid cancer	200 μCi	Weight loss and ^131^I enter the internal circulation	Constant weight	[96]
	Dexmedetomidine (3 μg/kg)	Thirty-six Wistar albino female rats (n = 12)	111 MBq	MDA, advanced oxidized protein products↑, total sulfur group, CAT↓	MDA, advanced oxidized protein products↓; total sulfur group, CAT↑; liver protection	[97]
	Montelukast (10 mg/kg/day)	Fifty female Wistar albino rats (n = 10)	111 MBq/kg	Inflammation and pulmonary fibrosis	Reduced the degree of inflammation and pulmonary fibrosis	[45]

## Data Availability

Not applicable.

## Abbreviations

^131^iodine (^131^I); 8-Epi-prostaglandin F2alpha (8-epi-PGF2α); aberrant cells excluding gaps (CAEG); anti-reactive oxygen metabolites (Anti-ROMs); American Thyroid Association (ATA); binucleated cells that present MN (MNCB); catalase (CAT); chromosome aberrations (CA); differentiated thyroid carcinoma (DTC); double-strand breaks (DSBs); excretion fraction (EF); excretion ratio (ER); first-minute uptake ratio (FUR); glutathione (GSH); glutathione peroxidase (GPX); hypoxia inducible factor-1α (HIF-1α); keratinocyte growth factor-1 (KGF-1); micronucleus (MN); maximum uptake ratio (MUR); NADPH oxidase (NOX); nanoparticles (NP); nuclear factor-kappa-B (NF-кB); radioiodine (RAI); reactive oxygen metabolites-derived compounds (d-ROMs); recombinant human thyrotropin (rhTSH); sodium iodide symporter (NIS); superoxide dismutase (SOD); single-strand breaks (SSBs); thioredoxin (Trx); thyroid-stimulating hormone (TSH); total antioxidant capacity (TAC); total oxidative stress (TOS); uptake fraction (UF); uptake index (UI).
